# Impact of screen use on tear breakup time: associations with ophthalmological factors and break frequency in office workers

**DOI:** 10.5935/0004-2749.2025-0083

**Published:** 2025-09-10

**Authors:** Berire Şeyma Durmuş Ece, İremnur Altındaş

**Affiliations:** 1 Department of Ophthalmology, Kastamonu University Faculty of Medicine, Kastamonu, Turkey

**Keywords:** Tear film, Screen time, Tear breakup time, Office workers, Protective factors, Lacerations, Refractive errors, Risk factors

## Abstract

**Purpose:**

To examine how ophthalmological features, screen exposure duration, and break habits among office employees affect ocular surface parameters.

**Methods:**

This single-center cross-sectional study involved two assessments on the same day: one before and one after a visual display terminal task. During the initial assessment, information on screen use was gathered, and refractive error, anterior segment examination, tear breakup time, and Schirmer test measurements were conducted. Participants tracked their screen usage and break durations throughout the day. At the end of the workday, tear breakup time and Schirmer I tests were repeated. Baseline and follow-up results were compared, and regression analysis was performed to identify factors linked to tear breakup time reduction.

**Results:**

The study enrolled 60 female office employees. Their mean screen time was 269.26 ± 70.21 min, with an average break duration of 151.93 ± 46.24 min. Tear breakup time at the second assessment (6.38 ± 2.70) was significantly lower than at baseline (8.62 ± 2.73) (p<0.001), whereas Schirmer test scores showed no significant change (p>0.05). Tear breakup time reduction was noted in 54 participants (90.0%), with a significant association between tear breakup time decrease percentage and screen exposure (p=0.001, r=0.463). Regression analysis showed that uncorrected or undercorrected refractive error was an independent risk factor for a ≥30% tear breakup time reduction, while taking more frequent short breaks (<15 min) acted as a protective factor.

**Conclusions:**

Taking more frequent short breaks (<15 min) and correcting refractive errors help prevent intra-day tear breakup time decline during visual display terminal use. Structuring breaks to support tear film stability is advisable for occupations that require regular visual display terminal tasks.

## INTRODUCTION

Computer vision syndrome (CVS), also known as digital eye strain (DES), describes a group of eye and vision-related symptoms resulting from extended use of visual display terminals (VDTs) such as computers, tablets, e-readers, and smartphones. Typical symptoms of CVS include eye discomfort, blurred vision, double vision, asthenopia, excessive tearing, eye redness, temporary changes in color perception, and increased glare sensitivity. It can also cause extraocular symptoms like headaches, neck pain, and shoulder discomfort. Al though these symptoms are generally temporary, they may persist beyond the workday^(1^,^2)^.

The professional and recreational use of VDTs has steadily risen in recent years. Despite the many benefits of digital devices, prolonged VDT use can negatively affect vision and the ocular surface. Previous research has shown that individuals who use VDTs for more than 4–6 h daily have a higher incidence of CVS-related visual symptoms and associated dry eye disease (DED)^(3^,^4)^. Estimates suggest that about 64%–90% of computer users experience CVS symptoms, and a meta-analysis has reported the prevalence of DED related to CVS to range from 9.5% to 87.5%^(5^,^6)^. Studies have identified the main risk factors for CVS as extended VDT use, reduced blink rate, uncorrected refractive errors, accommodation problems, screen glare from surrounding light, low ambient humidity (<40%), and poor ergonomic conditions^(1)^. Screen use can lead to changes in the ocular surface and tear breakup time (TBUT), which may present as visual discomfort, fluctuating vision, and decreased work performance^(2)^.

Although previous studies have explored the impact of total screen time on ocular surface health, few have addressed the role of break duration and characteristics. This study aims to assess the effects of ophthalmological factors, screen time, and break patterns among office workers on ocular surface outcomes.

## METHODS

The study enrolled office workers aged 20–40 years with a spherical equivalent between −2.00 and +2.00 diopters (D), who performed tasks using desktop computers and volunteered to participate. Individuals over 40 years were excluded to avoid age-related factors such as presbyopia. Additional exclusion criteria included a history of refractive surgery, keratoconus, use of rigid gas-permeable or soft contact lenses, strabismus, any active ocular disease, systemic conditions that could influence ocular surface results, use of systemic medications known to cause dry eye (e.g., retinoic acid, antidepressants), a diagnosis of DED, use of artificial tears, or the presence of pterygium or pinguecula. Participants with incomplete required records were also excluded. All participants provided informed consent after receiving an explanation of the study procedures. The study received ethical approval (2024-KAEK-30) and was conducted in accordance with the principles of the Declaration of Helsinki.

Participants underwent examinations twice on the same day. The first assessment took place in the morning (09:00–09:30 a.m.) before starting office work (baseline assessment). During the visit, participants provided information about their use of glasses, blue light filters on screens or glasses, computer screen text size, screen brightness settings, and humidifier use. Refractive errors were measured, and any uncorrected or undercorrected refractive error greater than 0.50 D was recorded as present. Next, an anterior segment examination, TBUT mea surement, and Schirmer test were performed. For the anterior segment exam, the corneal staining component of the Ocular Staining Score (OSS)–part of the dry eye grading system from the Sjögren’s International Collaborative Clinical Alliance (SICCA)–was used to quantify corneal staining. After fluorescein dye instillation, the cornea was examined under cobalt blue light using a slit lamp, and the number of punctate epithelial erosions (PEE) was counted. Scoring was defined as follows: no PEE scored 0, 1–5 PEE scored 1, 6–30 PEE scored 2, and more than 30 PEE scored 3. An additional point was added if PEE were found within the central 4 mm of the cornea, if filaments were present anywhere on the cornea, or if confluent staining areas were detected^(7)^. TBUT was then measured three times, with the average recorded as the study value. The Schirmer test was performed without local anesthesia (Schirmer I) about 15 min after the TBUT measurement.

 Participants’ screen time and break durations were documented using paper forms specifically created for data collection. These forms contained predefined sections to maintain consistency, and clear instructions were given to participants to help reduce recording errors. After the initial examination, participants received the forms and were instructed to log their screen use and break times in minutes throughout the day. The second assessment took place at the end of the workday on the same day (5:00–5:30 p.m.). During this follow-up, the completed forms were collected from the participants. TBUT and Schirmer I tests were then repeated using the same procedures. Only data from each participant’s right eye were included in the study.

### Statistical analysis

Data analysis was performed using IBM SPSS Statistics Version 26.0. Descriptive statistics included the mean, standard deviation, frequency, and percentage values. The distribution of quantitative variables was evaluated using the Kolmogorov–Smirnov or Shapiro–Wilk test, depending on the sample size. Numerical data are presented as mean ± standard deviation (SD), and categorical data are shown as counts (n) and percentages (%). The chi-squared test was applied for comparisons of categorical variables, while the Mann–Whitney U test was used for comparing quantitative data between groups. The Wilcoxon test was used to compare TBUT and Schirmer test results between the baseline and the follow-up assessment within the same group. Spearman correlation analysis was conducted to explore the relationship between the percentage reduction in TBUT and screen time. The dependent variable was defined as the “presence of a TBUT decrease of 30% or greater” (positive/negative). Both univariate and multivariate regression analyses were carried out, with variables found significant in the univariate analysis included in multivariate regression to determine independent risk factors. The backward logistic regression method was used for the multivariate model. Results are reported as odds ratios (ORs) with corresponding 95% confidence intervals (CIs). A p-value of <0.05 was considered statistically significant for all analyses.

## RESULTS

A total of 60 female office workers participated in the study, with a mean age of 33.4 ± 5.4 years. Table 1 shows the demographic details, office environment conditions, and ophthalmological examination findings of the participants. Among them, 15 participants (25.0%) had un corrected or undercorrected refractive errors, and none had overcorrection. Fourteen participants (23.3%) re ported using a blue light filter (Table 1). Of these, eight par ticipants (13.3%) wore blue light filter glasses, while six participants (10.0%) used a blue light filter on their digital screens. Ocular surface staining was present in 17 participants (28.3%), while 43 participants (71.7%) showed no staining.

**Table 1:** Demographic data, work environment conditions, and ophthalmic features of the office workers

Characteristics	Office workers (n=60)
Age, years	
Mean ± SD	33.4 ± 5.4
Wearing glasses, n (%)	
Present	14 (23.3)
Absent	46 (76.7)
Uncorrected or undercorrected Refractive error, n (%)	
Present	15 (25.0)
Absent	45 (75.0)
Using blue light filter, n (%)	
Present	14 (23.3)
Absent	46 (76.7)
Computer screen font size	
Mean ± SD	21.3 ± 0.2
Computer screen brightness, %	
Mean ± SD	63.6 ± 18.7
SE, diopter	
Mean ± SD	−0.62 ± 0.75
Min-Max	−2.00/0.75
Ocular surface staining, n (%)	
Present	17 (28.3)
Absent	43 (71.7)
SICCA OSS	
Mean ± SD	0.57 ± 0.87
Min-Max	0-3

SD= standard deviation; SE= spherical equivalent; SICCA OSS= Sjögren’s International Collaborative Clinical Alliance Ocular Staining Score.

It was also observed that none of the participants used a humidifier in their workplace. The average screen time was 269.26 ± 70.21 min, and the average break duration was 51.93 ± 46.24 min.

**Figure 1:**
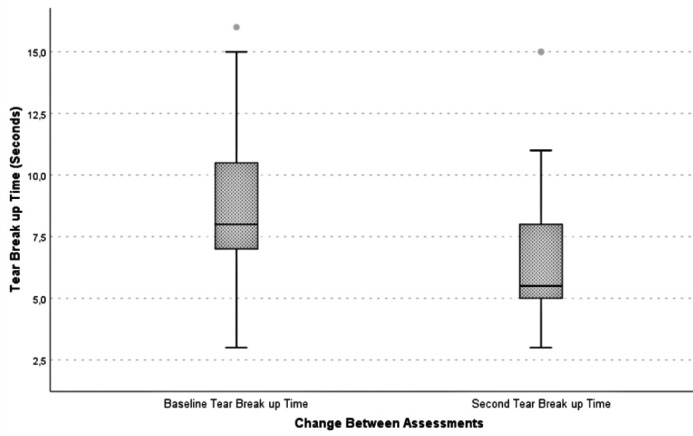
Box-and-whisker plot showing tear breakup time at baseline and at the second assessment.

**Figure 2:**
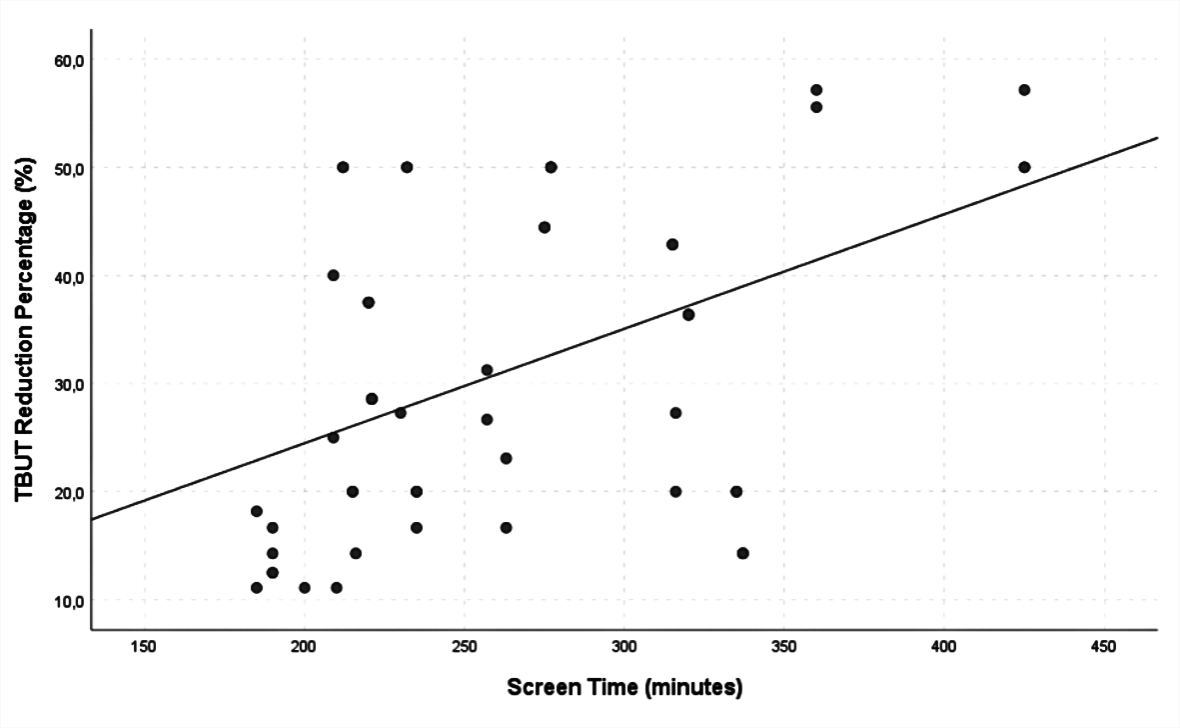
Correlation between screen time and the percentage reduction in tear breakup time (TBUT).

TBUT measurement were under 10 sec in 37 participants (61.7%) during the baseline assessment and in 53 participants (88.3%) during the second assessment. Table 2 presents the baseline and follow-up TBUT and Schirmer I test results. TBUT values at the second assessment (6.38 ± 2.70 sec) were sig ni ficantly lower than those at baseline (8.62 ± 2.73 sec) (p<0.001). The changes in TBUT are illustrated in figure 1. Only 6 participants (10.0%) showed an increase in TBUT at the second assessment compared to baseline, whereas a decrease was recorded in 54 participants (90.0%). Among those with a reduction in TBUT (n=54), a significant correlation was observed between the percentage decrease in TBUT and screen time (p=0.001, r=0.463) (Figure 2). There was no significant difference between the baseline and follow-up Schirmer test results (p>0.05).

**Table 2:** Tear breakup time and Schirmer I test results for the office workers.

Measurements	Office workers (n=60)		p-value^*^
	Baseline assessment	Second assessment	
TBUT, sec	8.62 ± 2.73	6.38 ± 2.70	**<0.001**
Schirmer I, mm	15.48 ± 6.34	14.43 ± 8.21	0.119

TBUT= tear breakup time.

* Wilcoxon test.

Participants were grouped categorized based on a 30.0% decrease threshold to control for possible daily variations in TBUT measurements (the median TBUT decrease percentage was 28.5%). Accordingly, those who showed a TBUT reduction of 30% or more at the second assessment (n=24) were designed as the positive group (Group 1) for TBUT reduction. In contrast, 30 participants with a decrease below 30% and 6 participants with an increase in TBUT were classified as the negative group (n=36) (Group 2) for TBUT reduction. Table 3 shows the comparison of digital screen use and break patterns between the groups. In Group 1, both total screen time and the number of work intervals longer than 45 min were significantly higher (p=0.031, p=0.012, respectively). Although total break duration did not differ significantly between the groups (p>0.05), Group 1 had significantly fewer breaks (p=0.006). The number of long breaks (≥15 min) did not differ between groups, but Group 2 had a significantly higher number of short breaks (<15 min) (p=0.034).

**Table 3:** Digital screen use and break durations of the office workers

Screen time and break characteristics	≥30% decrease in TBUT		p-value^†^
	Positive (Group 1) (n=24)	Negative (Group 2) (n=36)	
Screen time, min	290.83 ± 69.35	252.69 ± 63.08	**0.031**
Number of work periods with screen time ≥45 min	2.58 ± 1.21	1.69 ± 1.14	**0.012**
Breaks characteristics			
Break time, min	143.42 ± 41.17	158.69 ± 46.51	0.109
Number of breaks	6.46 ± 2.02	9.14 ± 4.05	**0.006**
Breaks by time			
Number of short breaks (<15 min)	4.08 ± 1.86	6.22 ± 3.81	**0.034**
Number of long breaks (≥15 min)	2.37 ± 1.28	2.94 ± 1.17	0.108

TBUT= tear breakup time; VDT= visual display terminal.

† Mann–Whitney U test.

Univariate and multivariate regression analyses were performed to assess the effects of various factors on the likelihood of a ≥30% reduction in TBUT. The univariate analysis showed a significant association between a ≥30% TBUT reduction and the presence of uncorrected or undercorrected refractive errors, total screen time, the number of work sessions lasting 45 min or longer, the total number of breaks, and the number of short breaks. In the multivariate analysis, uncorrected or undercorrected refractive errors were identified as an independent risk factor that increases the likelihood of TBUT reduction, while the number of short breaks was found to have a significant inverse association with TBUT reduction (p=0.012, OR=5.037; p=0.002, OR=0.829, respectively) (Table 4).

**Table 4:** Impact of variables on the likelihood of a ≥30% reduction in tear breakup time

	Univariate		Multivariate	
	OR (95% CI)	p-value	OR (95% CI)	p-value
Age	1.001 (0.909–1.102)	0.984		
Wearing glasses	0.789 (0.228–2.730)	0.709		
Uncorrected or undercorrected refractive error	4.429 (1.275–15.385)	**0.019**	5.037 (1.427–17.783)	**0.012**
Ocular surface staining	1.500 (0.482–4.669)	0.484		
Using blue light filter	0.520 (0.142–1.904)	0.323		
Computer screen font size	3.645 (0.372–35.728)	0.267		
Computer screen brightness	0.948 (0.956–1.012)	0.247		
Screen time	1.009 (1.000–1.017)	**0.039**		
Break time	0.992 (0.980–1.004)	0.192		
Number of work periods with screen time ≥45 min	1.928 (1.169–3.180)	**0.010**		
Number of breaks	0.742 (0.591–0.931)	**0.010**		
Number of short breaks	0.766 (0.609–0.963)	**0.023**	0.829 (0.734–0.937)	**0.002**
Number of long breaks	0.647 (0.431–1.054)	0.084		

## DISCUSSION

Extended use of VDTs for both work and leisure has become increasingly widespread. This trend places a large portion of the population at risk for CVS and prompts individuals to seek advise and treatment. Therefore, this study aimed to assess changes in ocular surface parameters among office workers performing VDT tasks and to identify factors influencing these changes by examining participants’ ophthalmological characteristics, screen time, break duration, and num ber of breaks.

In our study, we observed a significant reduction in average TBUT measurements among office workers after completing VDT tasks. Additionally, there was a significant correlation between the percentage decrease in TBUT and total screen time. The ocular surface changes linked to longer screen exposure have been attributed to several mechanisms. Reduced blink rates during extended screen use are a known contributing factor. Patel et al. reported a fivefold drop in blink rates during VDT use and demonstrated a connection between precorneal tear film stability and blink frequency^(8)^. Light emitted from VDTs and inflammatory processes are other mechanisms thought to play a role^(2)^. Prior studies have also shown that increased screen time is associated with a greater incidence of meibomian gland dysfunction (MGD), goblet cell dysfunction, and decreased mucus secretion^(9^,^10)^. Wu et al. found a correlation between MGD scores and VDT working hours in their research. They also reported that MGD presence was linked with increased corneal staining and more rapid TBUT^(9)^.

In our study, TBUT was measured twice at different times of the day. Previous studies examining the diurnal variation of TBUT have found that TBUT measurements are generally not influenced by the time of day^(11^,^12)^. Although the literature suggests that TBUT remains stable throughout the day, we classified participants with a 30% or greater TBUT decrease at the second assessment as the positive group for TBUT reduction to account for possible fluctuations. While there was no significant difference in break duration between our groups, the group with a significant TBUT decrease had a notably lower number of breaks. Our regression analysis showed that longer screen time, a higher number work intervals lasting 45 min or longer, and fewer breaks were linked to an increased chance of TBUT reduction. We also found that taking more frequent short breaks (<15 min) independently lowered the likelihood of TBUT reduction. Consistent with our findings, Al Dandan et al. found that infrequent breaks during screen use are an independent risk factor for DES^(13)^. Another study reported a significant relationship between fewer breaks during computer use and the onset of dry eye symptoms^(14)^. A common recommendation to counteract this is the 20-20-20 rule, which suggests taking a 20-sec break every 20 min to look at something 20 ft away. While some studies found that this rule did not affect tear film parameters, a randomized controlled trial reported that TBUT measurements were significantly higher after applying the 20-20-20 rule compared to baseline values^(15^,^16)^. Beyond their benefits for ocular surface health, studies have also shown that frequent breaks during screen work can improve employee productivity and well-being^(17)^. Our results indicate that taking short (<15 min) and frequent breaks during screen tasks, instead of long but infrequent ones, supports tear film stability and may help prevent CVS symptoms.

In our study, 25.0% of participants were found to have uncorrected or undercorrected refractive errors, which emerged as an independent risk factor for a greater reduction in TBUT at the second assessment. Achieving clear vision of small objects on a computer screen requires the image to be sharply focused on the retina. Therefore, even minor refractive errors (defined as 0.50 D in our study) can cause visual discomfort during VDT use, despite not affecting overall visual acuity. Previous research has shown that uncorrected astigmatism between 0.50 and 1.00 D can increase CVS symptoms^(18^,^19)^. Daum et al. estimated that appropriate refractive correction could boost productivity by at least 2.5%^(20)^. In occupations with extensive VDT use, correcting minor refractive errors–even those that do do not impact visual acuity–may help reduce constant accommodation demand and visual discomfort, thereby supporting tear film stability.

In our study, 61.7% of participants had a TBUT measurement below 10 sec at baseline, and 28.3% showed corneal staining. The average SICCA OSS among participants was 0.57 ± 0.87. An OSS of 3 or higher is considered abnormal^(7)^. Although the participants were asymptomatic and met the inclusion criteria, a significant portion of the group had baseline TBUT values under 10 sec, which could suggest subclinical tear film instability or undiagnosed DED. We did not observe a significant association between baseline corneal staining and TBUT reduction in our study. Nonetheless, preexisting ocular surface conditions and early-stage DED could contribute to the development of CVS. Our results might also have been affected by the mild degree of corneal staining found in participants or by the overlapping clinical characteristics of undiagnosed DED and CVS. This overlap may have influenced the TBUT reductions observed and could make it more challenging to attribute ocular surface changes solely to VDT use during the workday.

In our study, 23.3% of participants reported using a blue light filter while working on screens. Since electronic devices emit relatively low levels of blue light, the idea that blue light contributes to CVS remains debated^(1)^. We did not find a significant relationship between blue light filter use and TBUT reduction. Similarly, Singh et al. found that blue light blocking lenses did not affect signs or symptoms of eye fatigue compared to standard clear lenses during computer work^(21)^. However, other studies have suggested that blocking blue light may help alleviate CVS symptoms^(22^,^23)^.

This study has several limitations. One is that it did not account for environmental and ergonomic factors that might affect the development of CVS, such as screen distance, monitor height relative to eye level, poor lighting, and excessive glare. Although some studies have found no significant link between environmental conditions and DED in VDT users^(3)^, other research indicates that these factors can indeed play a role^(4)^. Another limitation is that the study only included participants aged 20–40 years. While this limits the generalizability of the findings to other age groups, it helped exclude individuals needing presbyopic correction, thereby removing the potential confounding impact of presbyopia.

Another notable limitation is that all participants were female. Some studies suggest that being female is an independent risk factor for CVS, which may limit how well these results apply to male populations^(3)^. Additionally, because all participants were female, the study did not control for possible effects of the menstrual cycle on tear film status, which should be considered in future research. Relying on self-reported screen and break times is another limitation, as this may introduce recall bias. While this study did demonstrate the benefit of more frequent breaks shorter than 15 min, it did not determine the minimum break length that would be effective for users, highlighting an area for further study. Other limitations include its single-center design, the use of only one PEE assessment, the evaluation being limited to a single day, and its cross-sectional nature, which does not allow for causal conclusions. Future longitudinal or interventional studies are needed to confirm these findings.

The choice of a ≥30% TBUT decrease as the cutoff for group comparisons was based on the median value found in our study and aimed to account for potential daily variations in TBUT. However, this threshold may not be applicable to other populations and should be interpreted cautiously.

In our multivariate logistic regression model, two variables–uncorrected or undercorrected refractive error and the number of short breaks–remained statistically significant predictors of a ≥30% TBUT reduction. The model achieved an event-per-variable (EPV) ratio of 12, which is above the commonly recommended minimum of 10 EPV needed for model stability. This suggests that the final model likely had sufficient statistical power to detect true associations. However, it should be noted that more candidate predictors were initially included in the variable selection process for the regression. Therefore, even though the final model met established statistical guidelines, the possibility of overfitting or reduced stability due to the small number of events at the initial stage cannot be completely dismissed. Future studies with larger and more diverse samples are needed to verify and expand on these results.

In conclusion, our findings indicate that taking more frequent breaks of less than 15 min and ensuring refractive errors are corrected are protective strategies against daily TBUT reductions related to VDT use. Regular eye exams to provide appropriate refractive corrections, along with the frequent scheduling of short breaks, are essential measures that should be adopted in work environments with routine VDT use to help maintain tear firm stability, prevent CVS, and promote individual well-being.

## Data Availability

The datasets produced and/or analyzed in this study can be provided to referees upon request.
